# Uterine Fibroids (Leiomyomata) and Heavy Menstrual Bleeding

**DOI:** 10.3389/frph.2022.818243

**Published:** 2022-03-04

**Authors:** Outi Uimari, Kavita S. Subramaniam, Beverley Vollenhoven, Thomas T. Tapmeier

**Affiliations:** ^1^Department of Obstetrics and Gynecology, Oulu University, Oulu, Finland; ^2^Research Unit for Pediatrics, Pediatric Neurology, Pediatric Surgery, Child Psychiatry, Dermatology, Clinical Genetics, Obstetrics and Gynecology, Otorhinolaryngology and Ophthalmology (PEDEGO) Research Unit and Medical Research Center Oulu, Oulu University Hospital, Oulu, Finland; ^3^St John's Institute of Dermatology, King's College London, Guy's Hospital, London, United Kingdom; ^4^Endometriosis CaRe Centre, Nuffield Department of Women's and Reproductive Health, University of Oxford, Oxford, United Kingdom; ^5^Women's and Newborn Program, Monash Health, Clayton, VIC, Australia; ^6^Department of Obstetrics and Gynaecology, Monash University, Clayton, VIC, Australia

**Keywords:** uterine fibroid, leiomyoma, heavy menstrual bleeding (HMB), somatic mutation, vascular architecture

## Abstract

Uterine Fibroids, or leiomyomata, affect millions of women world-wide, with a high incidence of 75% within women of reproductive age. In ~30% of patients, uterine fibroids cause menorrhagia, or heavy menstrual bleeding, and more than half of the patients experience symptoms such as heavy menstrual bleeding, pelvic pain, or infertility. Treatment is symptomatic with limited options including hysterectomy as the most radical solution. The genetic foundations of uterine fibroid growth have been traced to somatic driver mutations (*MED12, HMGA2, FH*^−/−^, and *COL4A5-A6*). These also lead to downstream expression of angiogenic factors including IGF-1 and IGF-2, as opposed to the VEGF-driven mechanism found in the angiogenesis of hypoxic tumors. The resulting vasculature supplying the fibroid with nutrients and oxygen is highly irregular. Of particular interest is the formation of a pseudocapsule around intramural fibroids, a unique structure within tumor angiogenesis. These aberrations in vascular architecture and network could explain the heavy menstrual bleeding observed. However, other theories have been proposed such as venous trunks, or venous lakes caused by the blocking of normal blood flow by uterine fibroids, or the increased local action of vasoactive growth factors. Here, we review and discuss the evidence for the various hypotheses proposed.

## Heavy Menstrual Bleeding and Uterine Fibroids

As many as 1 in 20 women aged between 30 and 49 years consult their GP each year because of heavy menstrual bleeding (HMB) or menstrual problems, with menstrual disorders the reason for 12% of all referrals to gynecology services in the UK (1). While HMB was historically given as a blood loss of more than 80 mL per day (2)—a definition not considered useful any longer given the large variation in women's physique and the fact that most women who seek treatment for HMB do not actually meet this criterion (3)—HMB is now defined as “excessive menstrual blood loss which interferes with a woman's physical, social, emotional and/or material quality of life” (1). It can occur on its own or in combination with other symptoms such as acute and chronic pelvic pain, or infertility (4). The severity can be estimated by self-reporting in questionnaires (5). The potential causes for HMB are many, such as ovulatory disorders, adenomyosis, endometriosis, endometrial polyps, and endometrial hyperplasia (6); however, the most common condition underlying HMB are uterine fibroids.

Uterine fibroids, or leiomyomata, are benign tumors of the myometrium arising within the uterus. Despite the name, fibroids largely comprise of myocytes rather than fibroblasts and are characterized by the excessive deposition of extracellular matrix substances, mainly collagen, within the tumor (7). The bulk growth of this extremely dense tissue leads an enlarged and deformed uterus and to some of the key symptoms associated with uterine fibroids in addition to HMB such as pressure symptoms, abdominal pain, and infertility (4, 6). In the United States, fibroids are cited to be the cause for over 50% of hysterectomies (8), and direct costs for their treatment is estimated between 4 and 9 billion USD (9).

Data on the incidence of uterine fibroids varies; while an Italian study of 341 non-care seeking women of reproductive age reported an incidence of 21.4% (10), a US study of 1,346 randomly selected women between 35 and 49 years screened by self-report, medical record and sonography, found an incidence of uterine fibroids by age 35 of 60% among African-American women, increasing to >80% by age 50, whereas Caucasian women in this study showed an incidence of 40% by age 35, and almost 70% by age 50 (11). An online survey of 21,479 women from Brazil, Canada, France, Germany, Italy, South Korea, the UK and the US on the other hand found a self-reported incidence of 4.5–17.8% in women of reproductive age (12), indicating the importance of sample population, age bracket and genetic background to reported susceptibility. In up to 40% of patients, uterine fibroids cause HMB (13), and more than half of the patients experience combinations of symptoms such as HMB, pelvic pain, or infertility (14, 15).

**Figure 1:**
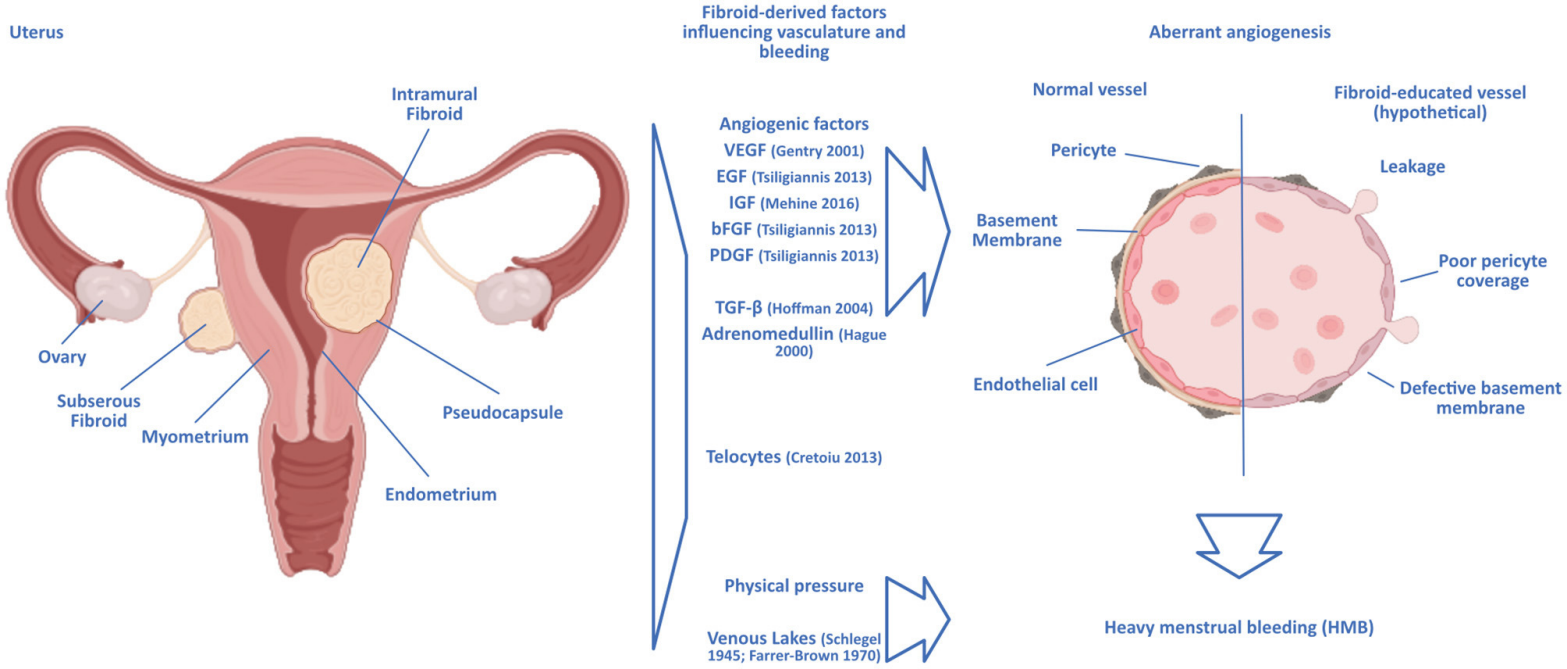
Hypothetical scenarios of a link between uterine fibroids and heavy menstrual bleeding. Intramural uterine fibroids are surrounded by a pseudocapsule of vasculature supplying the growing tumor. The growth and presence of fibroids can lead to aberrant angiogenesis similar to that observed in malignant solid tumors, with the chaotic formation of blood vessels in the vicinity of the fibroids. The vasculature surrounding fibroids would then be structurally deficient, rendering it prone to breaking and leakage. Angiogenic factors identified as expressed by fibroids are displayed. Other hypotheses focus on the connection with the formation of enlarged venous lakes within the endometrium, presumably induced by the mere physical pressure exerted by the fibroids. Ultrastructural analysis of fibroid-educated vasculature would help unravel the actual mechanisms at play.

Uterine fibroids are classified according to their location relative to the uterine anatomy in the FIGO system (16), but while intermenstrual bleeding as a symptom of uterine fibroids has been shown to correlate with the position and number of fibroids (13), the causal link to HMB is unknown. The classification of both HMB and the FIGO system are not without problems, as consistency between surgeons is lacking [Figure 1, (17)].

## Clinical Considerations and Treatment Options

With a comprehensive treatment plan lacking (6), current treatment of uterine fibroids largely provides symptomatic control. Treatment options are dictated by patient compliance, age, fertility preservation, and other common (co)morbidities such as endometriosis, adenomyosis, endometrial polyps, and endometrial hyperplasia that cause overlapping symptoms [pain and abnormal uterine bleeding (4)]. Follow-up evaluations of the tumor growth rate are recommended for asymptomatic fibroids by most evidence-based guidelines (18). Treatment options can be loosely classified by degree of invasiveness and the risk of reintervention (Table 1).

**Table 1:** Comparison of treatment options for uterine fibroid-related heavy menstrual bleeding.

**Treatment**	**FIGO type (16)**	**Preserves fertility**	**Reversible**	**Additional outcome**	**Treatment course**	**Reintervention rate** **within 5 years (19)**
NSAID	0–8	Yes	Yes	Pain relief	Long-term	N/A
TXA	0–8	Yes	Yes		Long-term	N/A
LNG-IUD	2–8	Yes	Yes	Pain relief	Long-term	N/A
CHC	2–8	Yes	Yes	Pain relief	Long-term	N/A
Progestin-only contraceptive	2–8	Yes	Yes	Pain relief	Long-term	N/A
UPA	1–8	Yes	Yes	Reduction of UF volume	Short-term	N/A
GnRH agonist	1–8	Yes	Yes	Reduction of UF volume	Short-term	N/A
GnRH antagonist	1–8	Yes	Yes	Reduction of UF volume	Short-term	N/A
UAE	1–6	Yes?	No	Reduction of UF volume	One-time intervention with long-term effects	14.4%
HIFU/MRgFUS	2–8	Yes	No	Reduction of UF volume	One-time intervention with long-term effects	53.9%
Hysteroscopic myomectomy	0–1/(2)	Yes	No	Removal of UF, definitive treatment	One-time intervention with long-term effects	7%
Laparoscopic/laparotomy myomectomy	3–8	Yes	No	Removal of UF, definitive treatment	One-time intervention with long-term effects	12.2%
Hysterectomy	2–8	No	No	Removal of uterus, definitive treatment	One-time intervention with long-term effects	0%

*NSAID, non-steroidal anti-inflammatory drugs; LNG-IUD, levonorgestrel-releasing intrauterine devices; CHC, combined hormonal contraceptives; UPA, ulipristal acetate; GnRH, gonadotropin-releasing hormone; UAE, uterine artery embolization; HIFU/MRgFUS, high intensity focused ultrasound/magnetic resonance imaging-guided focused ultrasound; TXA, tranexamic acid (most evident contraindications are listed in the manuscript text)*.

## Medical Management

Non-steroidal anti-inflammatory drugs (NSAID) reduce heavy menstrual bleeding (20). Although trials included in Cochrane meta-analyses commonly excluded UF and despite controversial evidence (21, 22), NSAIDs are recommended as an alternative option for levonorgestrel-releasing intrauterine devices (LNG-IUD) to treat HMB in women with fibroids smaller than 3 cm in size (1). The antifibrinolytic agent, tranexamic acid preserves the fibrin matrix structure and is widely used to prevent and treat blood loss, and it has been shown to be effective in fibroid-associated HMB (23).

Intrauterine devices such as the 52 mg LNG-IUD reduces menstrual bleeding in women with fibroids by inducing endometrial atrophy (24–26). The reduction in blood loss is significant, with evidence of successful treatment of anemia and increase in ferritin and hematocrit levels (27). However, the risk of expulsion for the device in a uterus with fibroids is increased compared to a non-fibroid uterus [11 vs. 0–3% (27)], with higher risks again for uteri with large and multiple fibroids. An additional limitation of studies published thus far is that they do not further explore those fibroid cases that fail in their aim to reduce HMB. There is no evidence yet on what type of fibroids LNG-IUD act on long-term, thus avoiding surgery, and for which subtypes other treatment options should be primarily considered. Many studies show a significant drop-out rate of participants with no response to LNG-IUD, who require definitive treatment, i.e., a hysterectomy (28, 29). No data on the effectiveness of lower doses of levonorgestrel are yet available.

Combined hormonal and oral progestin-only contraceptives can be considered in the treatment of fibroid-associated HMB, although evidence is limited (30) and partly based on expert opinion (23). Again, these products can be an option for fibroids smaller than 3 cm in size (1).

Selective progesterone receptor modulators (SPRM) moderate progesterone activity (31). Ulipristal acetate (UPA) binds to the intracellular progesterone receptor and blocks the effects of progesterone; it is thus effective in reducing total fibroid and uterine volume but results in amenorrhea during treatment in most women (32). Several randomized clinical trials have been evaluated in a Cochrane Review, and according to moderate-quality evidence, UPA improves the quality of life and reduces menstrual blood flow more than placebo and leuprolide (31). Similar results on quality of life, pain and bulk symptoms, efficacy and tolerability of UPA have been reported from RCTs in recent years (33–35). An exception are submucosal fibroids, which are less likely to respond with an improved bleeding pattern. Additionally, UPA improves the women's quality of life, and fibroid-related bulk and pain symptoms (36). It can be used preoperatively or as short-term management (usually as 3-month intermittent courses). Due to cases of serious liver injury that an UPA product (Esmya) was suspected of causing, the European Medicines Agency (EMA) has recommended periodic liver monitoring before, during, and after treatment with UPA in all prospective patients, to minimize any risk of developing liver failure (37). More data on UPA vis-à-vis surgical treatment for HMB and fertility outcomes in uterine fibroids are expected in the near future as several new trial protocols have been published recently (clinicaltrials.gov).

The anti-progestin mifepristone has been mainly studied and used for fibroid-associated HMB outside of Western countries. While its anti-glucocorticoid activity may limit its use, like UPA it decreases the size of fibroids, reduces heavy bleeding and improves pelvic pain symptoms and the quality of life, with spotting, elevations in liver enzymes, and endometrial hyperplasia reported as side effects (38, 39).

The estrogen receptor ligands raloxifene and tamoxifen act as selective estrogen receptor modulators (SERM). Their effect on fibroid size and associated symptoms have been investigated, but without significant evidence of alleviation of HMB (23).

Gonadotropin-releasing hormone (GnRH) agonists initially stimulate the pituitary gland and ovaries, then cause downregulation of GnRH receptors with full suppression of estradiol, causing a hypoestrogenic state. *Via* this mechanism they induce amenorrhea and reduce UF and uterine volume significantly but cause menopausal symptoms and bone loss (6). Due to these side effects, GnRH agonists are primarily used as short-course treatment (2–6 months) preoperatively to improve the effects of more conservative and less invasive surgical techniques (40).

GnRH antagonists competitively inhibit GnRH receptors in the pituitary gland and reduce circulating gonadotropins and ovarian sex hormones, including estradiol. Two oral GnRH antagonists with hormonal add-back therapies (indicated to offset hypoestrogenic effects including hot flushes, adverse lipid metabolism, and bone loss), elagolix and relugolix can be considered for the treatment of fibroid-associated HMB (41–43). Both have proven effective in reducing fibroid-associated HMB, and relugolix additionally seems to improve pain and bulk symptoms (43). In 2020, the U.S. Food and Drug Administration (FDA) approved the combination of elagolix (300 mg twice daily) with add-back therapy (1 mg estradiol and 0.5 mg norethindrone acetate once daily) to be used for up to 24 months (44). Relugolix (40 mg once daily) with add-back therapy (1 mg estradiol and 0.5 mg norethisterone acetate) was approved by the European Commission in July 2021 (45), and in October 2021 the agent was granted a license by the UK's Medicines and Healthcare Products Regulatory Agency (MHRA) (46).

## Radiological Management

Uterine artery embolization (UAE) can be recommended as a minimally invasive treatment for fibroid-associated bleeding and bulk symptoms in women who desire to preserve their uterus (47). An embolic agent is delivered through catherization of both uterine arteries to cause devascularization and involution of uterine fibroids. After UAE, significant reductions in fibroid and uterine volumes have been observed, which were maintained for up to 5 years (23). The bleeding pattern is usually improved following embolization, and the quality of life 2–5 years after treatment is similar among patients undergoing UAE, hysterectomy, or myomectomy (48). Although the surgical reintervention rate for UAE is higher than that for myomectomy [14.4 vs. 12.2% at 60 months (19)], rates for major post-procedural complications are lower in comparison to any type of surgery for uterine fibroids (19, 49).

Focused ultrasound procedures guided by diagnostic ultrasound (high intensity focused ultrasound, HIFU) or magnetic resonance imaging (Magnetic Resonance-guided Focused Ultrasound, MRgFUS), are non-invasive treatments using multiple high-intensity ultrasound waves to cause coagulative necrosis within fibroids (50). According to the limited published data, HIFU and MRgFUS reduce both fibroid and uterine volume (23) and improve the quality of life—but the evidence is of low quality (51). The rate of reintervention after HIFU is estimated to be as high as 53.9% at 60 months (19).

Radiofrequency ablation (RFA) of fibroids is a minimally invasive procedure that uses heat generated through radiofrequency waves with ultrasound guidance to induce coagulative necrosis in targeted fibroids to reduce their size. RFA can be delivered *via* a laparoscopic, transvaginal, or transcervical approach depending on fibroid location. UF volume reductions have ranged from 32 to 66% at 12 months of follow-up, and at 77% at later time points. RFA improves UF associated symptoms and quality of life. Surgical reintervention rate at 3 years is 11.5% (50). While RFA thus seems to be a good management option for symptomatic fibroids, access to this procedure is currently very limited. Both of these methods rely on adequate imaging to guide treatment.

## Surgical Management

Endometrial ablation is a procedure performed *via* hysteroscopy that surgically destroys a layer of endometrium to reduce menstrual bleeding. Current evidence is insufficient to assess the effectiveness of this management option to improve fibroid symptoms (23).

Myomectomy describes the surgical removal of fibroid tissue either *via* hysteroscopy, laparoscopy, laparotomy, minilaparotomy, or laparoscopically-assisted minilaparotomy. Hysteroscopic myomectomy is the primary management option for HMB from submucosal (FIGO type 0) and partly submucosal fibroids (FIGO type 1, ≥50% of the fibroid situated within the uterine cavity). Myomectomy can also be considered for FIGO type 2 fibroids (partly submucosal fibroid with ≥50% in an intramural location), albeit with a higher risk for repeated surgery or further need of medical management. Hysteroscopic myomectomy significantly improves fibroid-associated symptoms and quality of life, and the reintervention rate is as low as 7% at 5 years (19). Myomectomy *via* laparoscopy or laparotomy are a second-line treatment for FIGO type 3–8 fibroids if medication has failed (6, 50). Improvements in the quality of life are similar regardless of surgery type, but laparosopic myomectomy is associated with faster recovery time (50). The surgical reintervention rate for laparoscopic and laparotomy myomectomy is 12.2% (19).

The definitive surgical management for the treatment of fibroid-associated HMB, pain and pressure symptoms is hysterectomy. It is suitable for women who do not desire future pregnancies or do not wish to retain their uterus. Hysterectomy substantially improves hemoglobin levels and anemia, bulk symptoms (23, 52, 53) and the women's quality of life (54). However, blood transfusions following intraoperative hemorrhage, thromboembolism, and intraoperative bowel/bladder/ureter injury are apparent risks (23). The size and shape of the uterus (deformed by fibroids) directs the chosen hysterectomy route (vaginal, laparoscopic, and laparotomy).

The analysis of data from uterine fibroid registries such as COMPARE-UF in the United States could provide comparative effectiveness data regarding treatment options in the future (55).

## Angiogenesis in Uterine Fibroids

Hardly any tissue in the human body undergoes angiogenesis to the degree and frequency as the endometrium, where angiogenesis is vital during the growth of the endometrium in the proliferative phase of the menstrual cycle, the build-up and elongation of spiral arteries in the secretory phase, and the repairs during and after menstruation (56). In the neighboring myometrium, angiogenesis has been shown to be influenced by the presence of uterine fibroids through an increased proliferative response to estrogen and progesterone in smooth muscle cells in the presence of fibroids compared to normal myometrium [Figure 1, (57)].

An array of angiogenic factors involved in the vascularization and growth of uterine fibroids has been identified, including epidermal growth factor (EGF), heparin-binding-EGF, vascular endothelial growth factor (VEGF), insulin-like growth factor (IGF), basic fibroblast growth factor (bFGF), platelet derived growth factor (PDGF), transforming growth factor-β (TGF-β), and adrenomedullin (58). Driver mutations within uterine fibroids have been elucidated in the past two decades as a mutated Mediator Complex subunit 12 (MED12), mutations in the gene encoding the DNA-binding high mobility group AT-hook 2 (HMGA2), fumarate hydratase (FH) deficiency, and mutations in the genes encoding the collagen type IV α5 chain/collagen type IV α6 chains (Col4A5/A6) (59). Interestingly, 65% of tumors show mutations in the *MED12* gene (60), and another 25% show aberrations in *HMGA2*-driven gene expression (15, 61). Uterine fibroids arising from these two mutations show different characteristics; with fibroids driven by *MED12* mutations more numerous but smaller in size (62) compared to fibroids driven by *HMGA2* (63). MED12 has been shown to alter WNT/β-catenin pathway expression (64). In a mouse model of uterine fibroids, this resulted in a breakdown of cytoplasmic and an increase in nuclear levels of β-catenin, associated with an increased fibroid burden (65). Knockdown of the *MED12* gene on the other hand resulted in decreased proliferation of fibroid cells as induced by the WNT/ β-catenin pathway, and thus decreased fibroid growth (66). Apart from growth-promoting effects, these mutations increase the expression of downstream targets including angiogenic factors, most notably the insulin-like growth factor (IGF) system (59). The factors of the IGF family and their binding proteins (IGFBPs) have been shown to regulate tube formation and cell migration in endothelial cells (67), and could explain the formation of blood vessels around the uterine fibroids in the absence of HIF-1α/VEGF signaling.

The role of vascular endothelial growth factor (VEGF) has been studied in detail following its discovery in 1989 (68), and it has become one of the most important targets in anti-angiogenic tumor therapy (69). In uterine fibroids, VEGF levels have been reported as either similar (70) or increased (71) in fibroid tissues vs. adjacent myometrium, with an observation of declining VEGF levels after hysterectomy (72). Compared to myometrium, the expression of EGF was also reported to be higher in fibroid tissue (73), as did FGF (73), PDGF (73), TGF-β (74), IGF-1 (75), and adrenomedullin (76). An indication that angiogenesis in uterine fibroids could follow a different trajectory from the normal hypoxia response—with HIF-1α stabilization and the expression of VEGF as a result, which in turn induces sprouting and outgrowth of endothelial cells nearby to establish a connection to vascular supply (77)—was the finding that despite their hypoxic state, uterine fibroids surprisingly show a down-regulation of key players of the normal hypoxic response, HIF-1α for example has been shown lacking in uterine fibroids, when it was readily shown in leiomyosarcomata (78, 79). Aberrant vasculature as seen in tumors shows a chaotic structure and is prone to leaking; thus, alternative angiogenesis mechanisms, e.g., primarily through IGF signaling, might explain the HMB seen in women with uterine fibroids (80).

## Pseudocapsule

While the fibroid mass itself is poorly vascularised, it can be surrounded by a highly vascularised pseudocapsule in intramural fibroids, a specialized layer of tissue between the tumor and the surrounding myometrium that contains the blood vessels needed to sustain the fibroid (81), which develops in response to the fibroid growth (82). This structure can be seen as a “ring of fire” in ultrasound Doppler imaging, and it is separated from the myometrium by a clear cleft, as observed in histological images. The bursting of the vessels contained within the pseudocapsule could explain the HMB observed in women with uterine fibroids, in which case the symptom should correlate with the position of the fibroids, as the pseudocapsule only develops around intramural fibroids; this seems to be the case (13). The pseudocapsule is made up of the same cell types and shows the same biologic structure as the neighboring myometrium (83); however, the vasculature of the pseudocapsule might harbor structural defects that rend it susceptible to breaking, leading to HMB.

Responsible could be “telocytes,” a comparably recent discovery in the interstitial myometrium (CD34^+^c-kit^+^PDGFRα^+^). Telocytes have elongated telopodes, podomers, and regional podoms that stretch into the surrounding tissue; the cells thus stay in physical contact with many other cell types (84). In the uterus, telocytes are in especially close contact with smooth muscle cells (85) and are speculated to coordinate myometric contractions. Telocytes in the uterus express estrogen and progesterone receptors and are thought capable of regulating smooth muscle cell proliferation (84). Intriguingly, telocytes are not present within uterine fibroids but are found within surrounding myometrium (86). They express VEGF and could thus contribute to angiogenesis in the pseudocapsule.

## Venous Lakes

An older, classic theory of how HMB is connected to uterine fibroids suggests that “venous lakes” are responsible for the increased bleeding during menstruation: These large sinusoidal structures have long been known to form physiologically within the uterine vasculature from arteriovenous anastomoses (87). The blood flow into the venous lakes was thought to lead to a loss of pressure in the capillary system supporting the build-up of endometrium until the increase in venous lakes and the limited potential for increasing the supply pressure meant that the endometrium would die off, thus starting menstruation. Because the venous lakes lack any closing mechanism, they would bleed until completely sloughed off, and the menstrual loss of endometrium would only stop once the basal layer, supported by capillaries but without any arteriovenous anastomoses, had been reached. Microradiographic studies in the 1970's supported the notion that HMB resulted not from the fibroid vasculature itself but rather from venous lakes dilated and enlarged by virtue of increased interstitial pressure from the growing tumors (88). Once corrosion cast microscopy methods became available, studies into vascular changes within the myometrium in the presence of uterine fibroids indeed found venous lakes enlarged in uteri bearing fibroids in comparison to normal uteri (89), and further support to the theory was lent by immunohistochemistry studies of cyclic changes in uterine vasculature showing dilated and disintegrating venous vessels in the upper functionalis layer of the endometrium (90), showing that menstrual blood was largely venous in origin. However, the notion that physical pressure was the main factor leading to enlarged venous lakes was challenged once molecular biological analyses of angiogenic factors were able to show that indeed growth factors were largely responsible for the enlargement of veins and venous lakes (91), and that the heavy bleeding resulted from a failure of the fibrin/platelet plugs formed in the coagulation cascade in closing these blood vessels of increased diameter successfully.

## Vascular Architecture, Aberrations

The decisive factor could be the aberrant architecture of the fibroid-educated vasculature. Vasculature growing rapidly around malignant tumors in response to hypoxia is highly irregular, with high tortuosity, shunting of vessels and non-patent ends. Instead of ordered layers of pericytes and α-smooth muscle actin, tumor vasculature lacks stabilization through pericytes, and the α-smooth muscle actin is merely sporadically wrapped around the vessels (92). This renders tumor vasculature leaky and prone to breaking, enables the intravasation of metastatic tumor cells and hinders the successful delivery of drugs to the tumor (93). The vasculature developing around uterine fibroids could similarly show structural defects; however, elucidating these requires microscopy-driven avenues of investigation rather than omics approaches, as the latter would not necessarily flag any difference in vascular architecture as long as the quantities of the building blocks are not altered significantly. If proven to be similarly defective, the architecture of fibroid-educated vasculature could be targeted in analogy to the vascular normalization angle in tumor therapies (94).

## Physical Mechanisms

Fibroids may cause HMB merely physically, i.e., through an increase in endometrial surface due to underlying fibroid growth; through an influence on normal myometrial contractility patterns; through an ulcerated or degenerating fibroid, or through uterine venous ectasia due to compression from the fibroids (95). Fibroids were shown to secrete increased levels of transforming factor-beta 3 (TGF-β3) in response to steroids, as the estrogen and progesterone native to the uterus (96). TGF-β is associated with fibrotic disease, such as renal (97) or pulmonary fibrosis (98), where it induces the emergence of collagen-producing myofibroblasts *via* an epithelial-mesenchymal transition (EMT) (97). Although distinct from fibrotic disease, a role of TGF-β in uterine fibroids may enhance our understanding of the pathomechanism leading to HMB. TGF-β3 secreted by fibroids has been shown to induce BMP-2 resistance in endometrium by down-regulation of BMPR-2, likely causing defective endometrial decidualization (99). TGF-β3 also reduces expression of plasminogen activator inhibitor-1 (PAI-1), Antithrombin III (ATIII), and thrombomodulin in endometrium, likely contributing to bleeding (99). Interestingly, seasonally different patterns of circulating levels of interleukins (IL)-10, IL-13, and IL-17—all associated with fibrotic disease—have been identified in women with fibroids, similarly pointing toward an involvement of the wound healing immune response in fibroid growth and possibly HMB (100).

## Conclusion

Uterine fibroids are one of the main indications for heavy menstrual bleeding, a symptom that causes considerable impairment to patients' quality of life. Apart from hysterectomy, none of the currently available treatment options addresses this problem satisfactorily, a conundrum underlined by the fact that many women require definitive surgical management after exhausting their medical management options. Treatment options that would go beyond the merely symptomatic will depend on further knowledge of the influence of uterine fibroids on blood vessel growth and structure. The somatic mutations giving rise to uterine fibroids hold some clues as to their angiogenic potential, but the causal link between uterine fibroids and heavy menstrual bleeding is not yet known. While several hypotheses have been proposed during the last decades, structural studies of the vascular architecture of the blood vessels supplying uterine fibroids and the specific angiogenesis mechanisms that lead to their growth are needed to unravel the causal link between uterine fibroids and heavy menstrual bleeding, and thus enable the search for better therapies.

## Author Contributions

TT and OU conceived of the idea. OU, KS, BV, and TT wrote and discussed the manuscript. All authors agreed to the final version.

## Conflict of Interest

The authors declare that the research was conducted in the absence of any commercial or financial relationships that could be construed as a potential conflict of interest.

## Publisher's Note

All claims expressed in this article are solely those of the authors and do not necessarily represent those of their affiliated organizations, or those of the publisher, the editors and the reviewers. Any product that may be evaluated in this article, or claim that may be made by its manufacturer, is not guaranteed or endorsed by the publisher.
